# Numerical Investigation into GFRP Composite Pipes under Hydrostatic Internal Pressure

**DOI:** 10.3390/polym15051110

**Published:** 2023-02-23

**Authors:** Tamer Ali Sebeay, Azzam Ahmed

**Affiliations:** 1 Engineering Management Department, College of Engineering, Prince Sultan University, Riyadh 11586, Saudi Arabia; 2 Mechanical Design and Production Department, Faculty of Engineering, Zagazig University, Zagazig, Sharkia, Egypt; 3 Department of the Textile Engineering, College of Engineering and Technology of Industries, Sudan University of Science and Technology, Khartoum, Sudan; 4 Safat College of Science and Technology, HG6V+RP2, Khartoum, Sudan

**Keywords:** hydrostatic internal pressure, GFRP pipe, failure modes, winding angles, finite element analysis, deformation

## Abstract

Glass-fiber-reinforced plastic (GFRP) composite pipes are used extensively in high-performance applications, due to their high stiffness and strength, corrosion resistance, and thermal and chemical stability. In piping, composites showed high performance due to their long service life. In this study, glass-fiber-reinforced plastic composite pipes with [±40]_3_, [±45]_3_, [±50]_3_, [±55]_3_, [±60]_3_, [±65]_3_, and [±70]_3_ fiber angles and varied pipe wall thicknesses (3.78–5.1 mm) and lengths (110–660 mm) were subjected to constant hydrostatic internal pressure to obtain the pressure resistance capacity of the glass-fiber-reinforced plastic composite pipe, hoop and axial stress, longitudinal and transverse stress, total deformation, and failure modes. For model validation, the simulation of internal pressure on a composite pipe installed on the seabed was investigated and compared with previously published data. Damage analysis based on progressive damage in the finite element model was built based on Hashin damage for the composite. Shell elements were used for internal hydrostatic pressure, due to their convenience for pressure type and property predictions. The finite element results observed that the winding angles from [±40]_3_ to [±55]_3_ and pipe thickness play a vital role in improving the pressure capacity of the composite pipe. The average total deformation of all designed composite pipes was 0.37 mm. The highest pressure capacity was observed at [±55°]_3_ due to the diameter-to-thickness ratio effect.

## 1. Introduction

Composite pipes are used extensively in industries. Multi-layered, filament-wound composite structures have several advantages, including high stiffness and strength, corrosion resistance, and thermal stability [1,2,3]. Therefore, as manufacturing technology has developed, there has been growing interest in applying fiber-reinforced composite pipes to benefit from their potential to replace steel pipes. The energy industry has adopted composite pipes into more applications where metals corrode or where a light weight is required [4]. These composite pipes are well known and referred to as glass-reinforced epoxy (GRE) [5,6,7]. The differences in manufacturing methods are critical to the composite pipe’s strength property and final application. Typically, steel has been used in piping applications [8], which provides good performance, especially under heavy mechanical loading (e.g., high pressure, significant pipe movement, etc.). However, steel pipes undergo degradation in aggressive environments because of internal or external corrosion and initial leakage [9], which can generate partial or total failure. Recently, a few studies focused on using new resistant and noncorrosive materials such as glass- and carbon-fiber-reinforced plastic composites.

Fiberglass-reinforced polymer (FRP) is widely utilized in various applications due to its superior performance, including applications in pipes for oils and gas [10,11,12,13], bridge engineering [14,15], public and industrial buildings [16], marine construction [17,18,19,20], and underground infrastructure. Three types of commercially available materials (glass-fiber-reinforced polymer (GFRP), carbon-fiber-reinforced polymer (CFRP), and aramid-fiber-reinforced polymer (AFRP)) can be readily applied in a pipe used for energy applications. Compared to steel, FRP materials are insensitive to chloride-induced corrosion due to their non-metallic and noncorrosive intrinsic properties, which can significantly improve the corrosion resistance of the structure. Steel corrosion is a significant cause of the loss of hermeticity in oil and gas pipelines, and it is challenging to replace this material with non-corrosive materials [21,22].

The physical and mechanical properties of the FRP materials are shown in Table 1, as well as traditional steel. Compared to steel, FRPs have a lighter weight and higher strength. However, their mechanical properties are linear elastic with no prominent yielding stage, which leads to a lower failure strain and elongation rate. In addition, the FRP materials’ Young’s modulus is usually lower than steel’s (except for some CFRPs with a high elastic modulus).

In the energy Industry, there are two distinct manufacturing processes of composite pipes: filament wound and centrifugal casting [24]. The similarities are the fiberglass strands or other materials that can be wrapped around or in a mold to create strong pipes and the fact they are engineered with characteristics that overcome weaknesses in metals [25]. Metallic structures tend to require more inspection, repair, and maintenance during their service life, meaning a need to schedule shutdowns while increasing expenditure. These shutdowns are mitigated against in composite pipes as they remove corrosion-related problems and the need for corrosion inhibitors or cathodic protection. With a lack of scaling and other bore restriction issues in composite pipes, they also have a superior internal fluid flow performance compared to metallic pipes. Composite pipes also demonstrate super strength and stiffness with much less weight, making them easier to handle without lifting equipment, and reducing their lifecycle, transportation, and installation costs. As a result, they are used for oil and gas transportation, inshore and offshore, in chemical industries, drainage, and sewage systems, and for drinking water. Composite pipes wound with filament are also used in the aerospace, automotive, marine, construction, and sports sectors. Additionally, the composite pipes could be used in electric power [26], environmental protection [27], and other fields.

The effects of the winding angle on the behavior of glass/epoxy composite tubes under multiaxial cyclic loading were investigated. Glass-fiber-reinforced epoxy (GRE) composite pipes with three winding angles, namely [±45°]_4_, [±55°]_4_, and [±63°]_4_, were tested. The results indicate that each winding angle dominates a different optimum pressure loading condition, namely [±55°]_4_ for pure hydrostatic loading, [±45°]_4_ for the hoop to axial loading, and [±63°]_4_ for the quad hoop to axial loading [28]. To test the impact on pipes, four different stacking sequences were tested under impact after being internally pressurized [11]. The pipes were manufactured using filament winding with winding angles of [±45/±45/±45], [±55/±55/±55], [±63/±63/±63], and [±63/±45/±55]. Under internal pressure, the maximum capacity was 56 bars and this was recorded for the pipes with [±55]_3_ winding angles [11]. A comprehensive review of burst, buckling, durability, and corrosion analysis of lightweight FRP composite pipes and their applicability was reviewed by Prabhakar et al. [4]. The results showed that burst analysis revealed that the winding angle of ±55° was observed to be optimum with minimum failure mechanisms, such as matrix cracking, whitening, leakage, and fracture. The reduction of the buckling effect was reported in the case of the lower hoop stress value in the hoop-to-axial stress ratio against axial stress, compression, and torsion.

Numerical and experimental investigations of the hydrostatic performance of fiber-reinforced tubes were conducted by Pavlopoulou et al. [29]. Finite element (FE) models of the tubes were generated to analyze the theoretical performance of a more extensive range of tube thickness and internal diameters. The work concluded that the failure mechanisms of composite tubes when exposed to external hydrostatic pressures are complex. Therefore, design against external pressures needs careful consideration. A parametric study investigated the effect of fiber volume fractions and winding angles on the failure pressures of GFRP pipes subjected to internal hydrostatic pressure [29]. The study adopted five different volume fractions of 52.5%, 55%, 57.5%, and 60% and three different winding angles of 52.5°, 57.5°, and 60.19°. The results show that the functional and first-ply failure pressures decrease with increasing fiber volume fractions, while the higher winding angles enhanced functional failure pressures [30]. The characteristic behavior of the ±55° winding angle GRE pipe tested under multiaxial ultimate elastic wall stress (UEWS) tests was investigated by Pranesh et al. [31]. The investigation showed that the UEWS test can be used as an alternative to requalifying the GRE pipes described in the ASTM 2992 standard document. Van et al. [32] numerically simulated the pipe-in-pipe systems installed on an uneven seabed. The results showed that the equivalent pipe section can be used for on-bottom roughness analysis and the free span assessment of fully bonded pipe-in-pipe systems. Roham et al. [33] simulated and analyzed functional failure in composite pipes subjected to internal hydrostatic pressure. A progressive damage model was developed considering the influence of a core layer incorporated to increase pipe stiffness. The effect of two main parameters, core thickness and the winding angles of cross plies, were investigated. It was observed that first-ply failure (FPF) and functional failure (FF) pressures increase linearly with the increasing core thickness. Numerical studies on the global buckling of subsea pipelines were investigated by Liu et al. [34]. Four numerical simulation methods based on the finite element method (FEM) program ABAQUS, i.e., the 2D implicit, 2D explicit, 3D implicit, and 3D explicit methods, were used to simulate pipeline global buckling under different temperatures.

Industrial-scale manufacturers developed short-term tests such as the ultimate elastic wall stress test as an alternative method to determine the long-term hydrostatic pressure of GFRP pipes [35]. Costa et al. [36] presented a long-term high-pressure hydrostatic test of a composite repair system for metallic pipes with one-in through-wall (Type-B) corrosion defects. The results suggested a considerable pressure fluctuation during the measurement cycle. GFRP composite pipes were developed and studied for hydrostatic pressure with different end cap thicknesses. These pipes were manufactured with varying designs of interplay using two woven glass layers (±54°/90°) at the top layer. The failure in the composite pipe occurred at 15 MPa after a time of 6 s at the lowest thickness [37]. Mistry et al. [37] theoretically investigated the suitable winding angle for glass FRP pipes under external pressure and axial compression. Under the hydrostatic pressure condition, the optimum winding angle was calculated as 80° using the finite element method. Similarly, the carbon FRP tube was subjected to biaxial stress by inducing axial load and internal pressure [38]. The filament winding technology was the most used methodology for composite pipe manufacturing [28,29,39,40,41,42].

Many studies have been implemented on the mechanical performance of GFRP thermoset composite pipes. Roham [43] studied stress/strain analysis, failure evaluation, environmental issues, viscoelastic behavior and creep analysis, fatigue analysis, and impact analysis. Gunoz et al. [44] investigated the hardness and density properties of GFRP composite pipes under seawater conditions. Rafiee et al. [45] evaluated the mechanical performance of GFRP pipes subjected to transverse loading. Tensile strength alterations in GFRP composite pipes under seawater-dominated conditions were investigated by Gunoz et al. [46]. A comprehensive experimental study on the mechanical characterization of particulate FRP composite pipes was implemented by Saghir et al. [47]. Hawa et al. [48] studied the burst strength of glass fiber/epoxy composite pipes subjected to impact loading. The results indicated that the peak force and contact time increase with the increased impact energy. The effects of accelerated hydrothermal aging on the behavior of composite tubes under multiaxial stress were experimentally investigated. A set of [±55°]_4_ tubes were hydrothermally aged at 80 °C for 1500 h [49]. One consideration for this study was using GFRP composite pipes instead of CFRP and AFRP pipes due to low cost of glass fibers, mechanical properties, widely used in pipes for a long time, and its adoption with code standardizations. In addition, glass-fiber-reinforced plastic (GFRP) pipes represent an attractive alternative to other pipelines subjected to severe internal or external environments in onshore or offshore applications due to their corrosion resistance properties, which reduce maintenance and costs and lengthen the lifetime of the pipe.

Table 2 summarizes the data found in the literature studying the internal and external pressure effect on the pressure capacity and the failure mode of composite pipes. Most of the available literature considered internal pressure, which is a fact due to the function of the pipeline. However, during their lifetime, pipes are subjected to external pressure, especially those pipes that are installed on the seabed and/or buried underground, for which our current paper fits. The objective of the current paper is to numerically check the effect of internal pressure on a composite pipe with different parameters such as varied winding angles, pipe thickness, and pipe length. Internal pressure simulates the hydrostatic pressure on the inner surface of composite pipes installed on the seabed and under deep soil.

## 2. Problem Statement

As was shown in the introduction, the problem of a composite pipe under internal pressure has not been investigated, considering all the macro- and micro-damage mechanisms, although it is a real case scenario. The consumption of tremendous amounts of raw materials, mainly steel, in pipe production has raised severe environmental concerns under the seabed or soil due to corrosion, failure, and bursting. To avoid such overexploitation of steel, composite pipes are studied, as an attractive alternative for energy tubes and pipeline applications. As per the literature, FRP-composite pipeline structures subjected to hydrostatic internal pressure have been reported in the literature using various concerns such as winding angles, failure modes, stresses, wall thickness, composite pipe radius, and optimum stacking sequence. This study aims to examine the stresses of GFRP composite pipes under a constant internal pressure of 10 MPa and check the effect of length-to-pipe diameter (L/D), diameter-to-thickness (D/T) ratio, and winding angles of the GFRP composite pipe.

This study investigates the failure behavior, hoop stress, axial stress, and total deformation of a GFRP composite pipe with varied winding angles, pipe lengths, and pipe thicknesses. The effect of constant pressure on the GFRP composite pipe structure is investigated, and commercial ABAQUS/CAE 2020 is used to obtain the simulation results and compare them with experimental results.

In an earlier study, glass-fiber-reinforced epoxy (GRE) pipes with an internal diameter of 110 mm, a length of 450 mm, and various wall thicknesses (3.78–6.3 mm) were fabricated using a wet filament winding process [11]. The GFRP composite pipe was exposed to internal pressure to determine its capacities and failure modes. ARALDITE LY-1564 epoxy resin, mixed with hardener, was used as matrix material. GFRP pipes were manufactured using a filament angle machine, with [±45]_3_, [±55]_3_, [±63]_3_, and [±63/±45/±55]_5_ winding angles. The details of the GFRP composite pipe fabrication and internal pressure testing can be found in our previous study [11]. The [±55]_3_ winding angle showed the best response under internal pressure, whereas the combined one was the best under impact.

## 3. Composite Pipe Modeling

Theoretical modeling was carried out to estimate the load-bearing capacity of the GFRP composite pipe and failure modes using Hashin damage criteria after being subjected to hydrostatic internal pressure. Abaqus commercial software was used to simulate the GFRP composite pipe.

**Figure 1:**
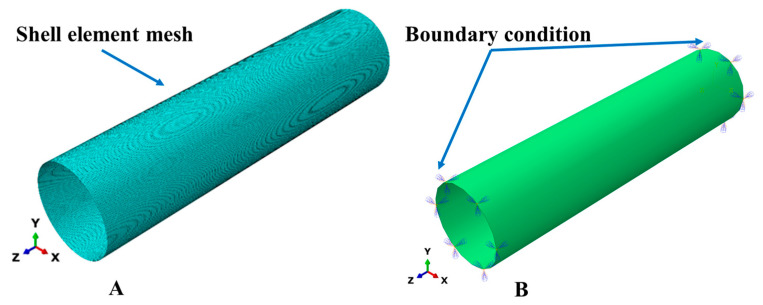
Illustration of the FE model. (**A**) meshed model, (**B**) boundary conditions.

**Figure 2:**
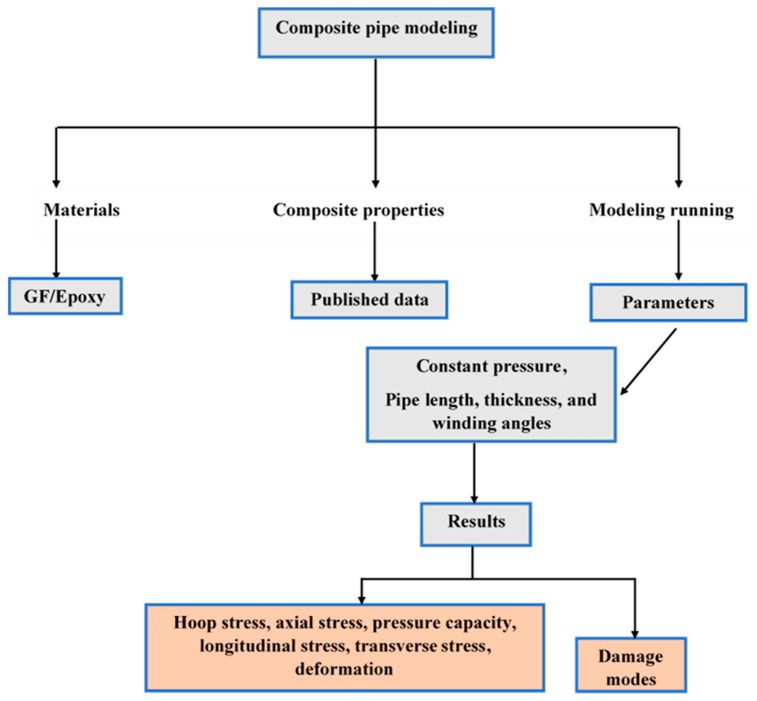
Flowchart of composite pipe modeling.

The finite element (FE) model of GFRP composite pipe was created in Abaqus commercial software 2020. S4R shell elements were used for the 18-pipe model. The model adopted the mesoscale, where the mechanical properties of each layer were fed to the model in the principal directions. Model mesh (1-A) and boundary conditions (1-B) were as shown in Figure 1. The flowchart of the composite pipe model is summarized in Figure 2.

**Figure 3:**
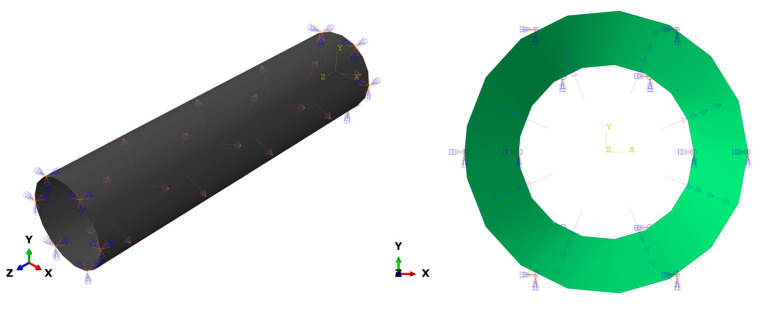
GFRP composite pipe subjected to hydrostatic internal pressure.

Symmetric/encastre boundary conditions were applied to the model at both ends of the GFRP composite pipe as fixed displacements and rotations. The type of the subjected load was pressure with hydrostatic distribution. A uniform internal pressure of 10 MPa was applied to the inner surface of the GFRP composite pipe model, as shown in Figure 3. The GFRP composite pipe lay-up sequence with different winding angles, pipe lengths, and pipe thickness was fed into the Abaqus. The mechanical properties of GFRP composite laminates are presented in Table 3. Geometrical nonlinearity was considered in the solutions steps.

The GFRP composite pipe was designed with a varied winding angle, from [±40]_3_ to [±70]_3_, and a different pipe length (110–660 mm) and thickness (3.78–5.1 mm). Simulations were considered for *n* = 3, which means the number of layers was 3 (See Table 4). This selection was because these staking sequences are the most common in the composite pipe industry. In addition, it is the optimum staking sequence for a thin-walled cylinder subjected to internal pressure [57]. A total of 18 GFRP composite pipe models with different parameters were carried out. The pressure capacity of each pipe was measured, as well as failure mode at constant internal pressure being discussed and compared with each other. The output of the simulations was hoop stress. Axial stress, pressure capacity, S. Mises stress, longitudinal and transverse stress, failure mode, and total deformation of the 18 different GFRP composite pipes. It is worth noting that the 3D assumption was adopted to capture the possible effect of the end clamp of the pipe.

## 4. Failure Inspection

Hashin criteria were adopted where multiple stress components were used to evaluate different failure modes. The Hashin criterion is usually implemented in the 2D classical lamination method, and the point stress calculation is performed with point conversion as the material degradation model. The criterion is extended to three-dimensional problems, where the maximum stress criterion is used for the transverse normal stress component. Hashin’s failure criteria were used to predict the damage initiation, including four damage initiation mechanisms to detect matrix and fiber under tension and compression failures. The failure modes included in Hashin’s criterion are as follows:

Tensile fiber failure for $\sigma_{11}\;\geq 0.0$:

(1) $${\left(\frac{\sigma_{11}}{X_{T}}\right)}^{2}+\frac{\sigma_{12}^{2}+\sigma_{13}^{2}}{S_{12}^{2}}\geq 1.0$$

Compression fiber Failure $\sigma_{11}<0.0$:

(2) $${\left(\frac{\sigma_{11}}{X_{C}}\right)}^{2}\geq 1.0$$

Tensile matrix failure $\sigma_{22}+\sigma_{33}>0.0$:

(3) $$\frac{{\left(\sigma_{22}+\sigma_{33}\right)}^{2}}{Y_{T}^{2}}+\frac{\sigma_{23}^{2}-\sigma_{22}\sigma_{33}}{S_{23}^{2}}+\frac{\sigma_{12}^{2}+\sigma_{13}^{2}}{S_{12}^{2}}\geq 1.0$$

Compression matrix failure $\sigma_{22}+\sigma_{33}<0.0$:

(4) $$\left[{\left(\frac{Y_{C}}{2S_{23}}\right)}^{2}-1\right]\frac{\sigma_{22}+\sigma_{33}}{Y_{C}}+\frac{{\left(\sigma_{22}+\sigma_{33}\right)}^{2}}{4S_{23}^{2}}+\frac{\sigma_{23}^{2}-\sigma_{22}\sigma_{33}}{S_{23}^{2}}+\frac{\sigma_{12}^{2}+\sigma_{13}^{2}}{S_{12}^{2}}\geq 1.0$$

where, *σ*_ij_ represents the stress component, and the tensile and compressive strengths of the laminate are represented by the subscripts *T* and *C*, respectively. The strength coefficients are *X_T_*, *Y_T_*, and *Z_T_* under tensile loading and *X_C_*, *Y_C_*, and *Z_C_* under compression loading. In addition, *S*_12_, *S*_13_, and *S*_23_ represent shear strengths in the respective main material directions. It can predict the onset of the failure accurately by Hashin failure criteria due to a consideration of the interaction between different in-plane stress components. Delamination failure mode as an interlaminar failure was not considered in this study, assuming that under internal and/or external pressure the material would suffer from intralaminar damage before delamination. The GFRP composite pipe was considered with a thin-walled structure due to the thickness-to-diameter ratio of GFRP composite pipes being 0.037–0.046, which is smaller than 0.1. The pipe was considered to fail when one of the failure modes, such as matrix cracking, reached one in the Hashin damage criteria. Then after that, the composite pipe would be out of work due to its leakage or bursting.

## 5. Results and Discussion

Three studies for composite pipe modeling were taken into consideration and included were the effect of winding angles, the effect of pipe length to diameter (L/D), and the effect of diameter to thickness (D/T).

In the case of the effect of the winding angle, seven models were tested with different winding angles ([±40]_3_, [±45]_3_, [±50]_3_, [±55]_3_, [±60]_3_, [±65]_3_, and [±70]_3_) at the same pipe thickness (3.78 mm) and length (450 mm) and at a constant hydrostatic internal pressure (10 MPa), to obtain hoop stress, axial stress, and pressure capacity, which were compared with other structures and discussed. In the case of the pipe length to diameter (L/D) effect, the composite pipe with the [±55]_3_ winding angle and the same thickness, but with varying pipe lengths (110, 220, 330, 450, 550, and 660 mm), was subjected to constant hydrostatic internal pressure to obtain hoop stress, axial stress, and pressure capacity. In the case of the length to thickness (L/T) effect, the composite pipe with the [±55]_3_ winding angle and the same pipe length (450 mm), but with varied pipe thicknesses (3.9, 4.2, 4.5, 4.8, and 5.1 mm), and subjected to constant hydrostatic internal pressure to obtain hoop stress, axial stress, and pressure capacity, was investigated and discussed.

The GFRP composite pipe was fixed from both ends and the pressure on the internal surfaces was applied. The readings of pipe specifications, hoop stress, axial stress, hoop-to-axial ratio, internal pressure, and pressure capacity are presented in Table 5. In addition, composite pipe specifications with different parameters and simulation results, including S. Mises stress, longitudinal stress (*S*_11_), and transverse stress (*S*_22_), are presented in Table 6.

To validate the current model, the results of the [±55]_3_, 3.78 mm thick, and 450 mm long pipe, which were obtained numerically, were compared to the experimental results obtained by Sebaey [11], and excellent agreement was found in terms of the internal pressure capacity. The sensitivity analysis was decided based on the experimental results.

### 5.1. Effects of Winding Angles

**Figure 4:**
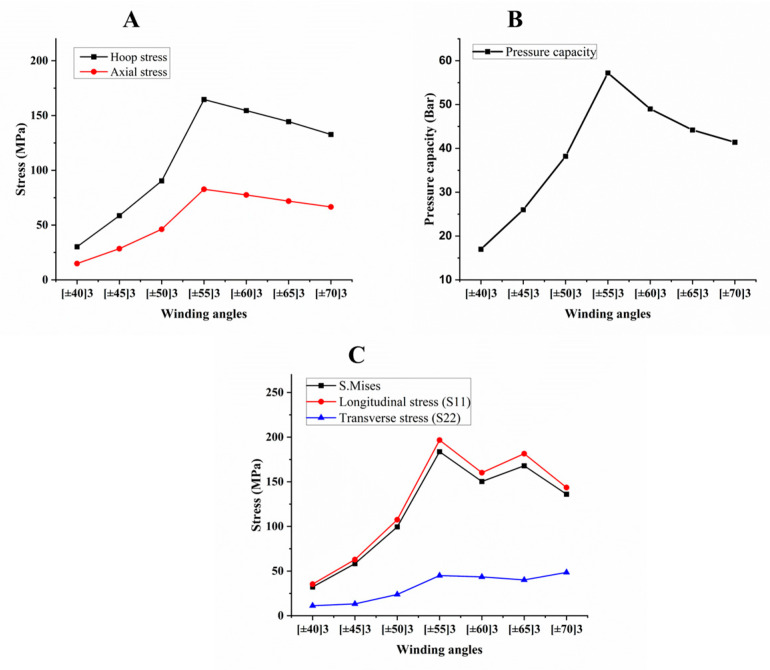
Hoop and axial stress (**A**), pressure capacity (**B**), and various stress (**C**) of composite pipe with different winding angles.

**Figure 5:**
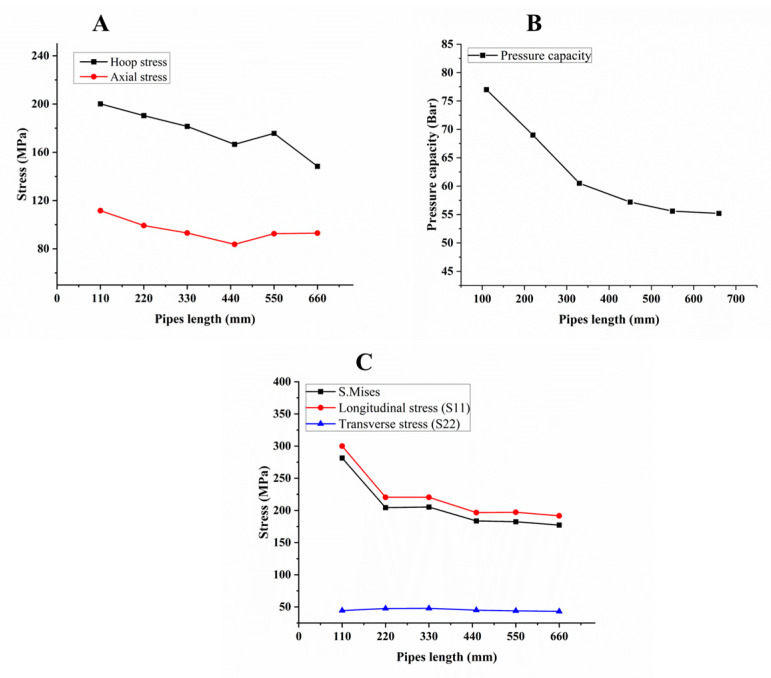
Hoop and axial stress (**A**), pressure capacity (**B**), and various stress (**C**) of [±55]_3_ composite pipes with different lengths.

**Figure 6:**
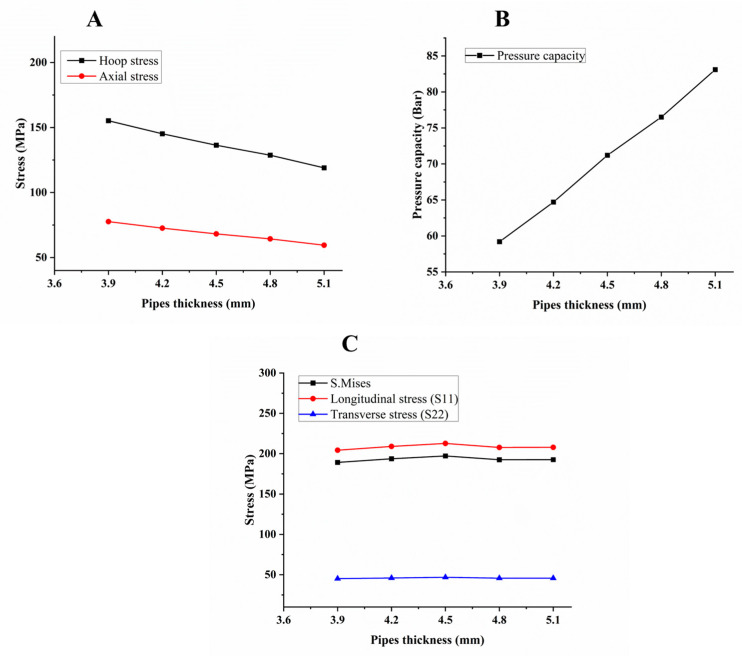
Hoop and axial stress (**A**), pressure capacity (**B**), and various stress (**C**) of [±55]_3_ composite pipes with different thicknesses.

From Figure 4, Figure 5 and Figure 6, hoop and axial stress, S. Mises, longitudinal and transverse stress, and the pressure capacity of the composite pipes were obtained and recorded from the model when the matrix cracking occurred in the structure and reached one, as seen in Table 6. In addition, comparisons between them are also discussed. Figure 4 explains the effect of winding angles when the composite pipe was subjected to constant internal hydrostatic pressure with various winding angles. Hoop and axial stress (4-A) showed an increase gradually up to [±55]_3_ and then decreased when the winding angle increased further, because of the reinforcement orientation and initial damage progress. The behavior of the structure with various winding angles exhibited nonlinear elastic behavior.

The pressure capacity in (4-B) shows an increase by increasing the winding angle up to [±55]_3_. S. Mises and longitudinal stress in (4-C) were shown to have the same track as pressure capacity up to [±60]_3_, and after that increased up to [±65]_3_, and then decreased. All properties showed the highest value at [±55]_3_, as per the literature; the optimum winding angle for the filament-wound pipe structure is 54.5° [58]. Sulu and Ismail [58] showed that the internal pressure capacity of a GFRP pipe is a function of the winding angles ([±52.5°]_n_, [±57.5°]_n_, and [±60.19°]_4_).

In the case of transverse stress, in (4-C), it is shown that the value of stress starting increasing from [±40]_3_ to [±55]_3_, decreased from [±55]_3_ to [±65]_3_, and then increased. Thus, this is attributed to the interface bonding strength of the structure with those winding angles, which leads to an increase in the transverse stress of the structure. In our previous studies in ref. [11], the experimental results of capacity bearing pressure for the [±55]_3_ winding angle were 56 bar, but in this study, the simulation results for the same specifications of the pipe, including diameter, length, and thickness, was 57.2 bar, as seen in Figure 4B. Thus, the numerical results show reasonably comparable trends with the experimental results and a similar evolution through the internal pressure results. The aspect differences between the two studies can be justified by the interaction between the individual layer in the filament winding that cannot be simulated.

### 5.2. Effect of Pipe Length to Diameter (L/D)

Figure 5 explains the effect of pipe length to diameter. Therefore, in our current case, the diameter of the pipe was constant at 110 mm, and the pipe length was variable (110–660 mm). Some of the predicted properties showed an inverse relationship; when pipe length increased and the L/D ratio increased, hoop stress, axial stress, S. Mises, longitudinal stress, and transverse stress decreased ((5-A) and (5-C)). However, in the case of pressure capacity (5-B), it was found that it decreased and the length effect should be ignored at a certain length, which means that the pressure capacity becomes stable after a certain length of the pipe. Thus, due to the vital and significant role of pipe length on the properties of the pipe, when the composite pipe has a long length, it may easily buckle when subjected to pressure if there is not enough support in the outer structure. The ratio of L/D should be taken into full consideration in pipe design. The behavior of the structure with various pipe lengths and the same wall thickness exhibited nonlinear elastic profile.

### 5.3. Effect of the Pipe Diameter to Thickness (D/T)

The pipe diameter–thickness ratio D/t is very important, in that it can determine the local critical collapse pressure of submarine pipelines [11]. In this study, the diameter of GFRP pipes was constant at 110 mm; however, the thickness varied from 3.9 to 5.1 mm. The ratio of D/t of the [±55]_3_ winding angle with a thickness of 5.1 mm was the lowest one compared with other structures. From Figure 6, the pipe thickness, among other effects shown, has a positive relationship trajectory in terms of pressure capacity compared to other structures. In this case, pressure capacity increased when thickness increased, due to the increased strength of the pipe within varied thicknesses, which then also led to increased axial stress, S. Mises, and longitudinal stress up to 4.5 mm. Transverse stress (6-c) had no significant effect, and then the value of the stress was almost the same due to the variation in pipe thickness not being large. The behavior of the structure with various wall thicknesses and the same winding angle exhibited linear elastic profile.

The effects of the decreased D/T ratio led to an increased pressure capacity. Sulu, Ismail, and Saeed [52,59] showed that the internal pressure capacity of a GFRP pipe is a function of pipe wall thickness or the number of layers, and the same results were obtained in the current study. The simulation results indicated that deformation and failure mechanisms depend on the winding angle. A comprehensive review of the finite element study of failure pressure estimation for an aged and corroded oil and gas pipeline was investigated by Velázquez et al. [60]. The main conclusion of this study was that failure pressure can be estimated more accurately by considering both corrosion defect characteristics and variations in the properties of the material caused by aging.

Maximum axial and hoop stresses occurred at a winding angle of 55°, as seen in Table 5 when comparing specimens with other angle-ply lay-ups.

As the experimental degradation process of the composite pipe when the internal pressure applied on the specimens starts to increase, the lengths of the specimens tend to shorten, while their diameters enlarge. As the pressure increases, whitening initiation is observed and this tendency keeps increasing. The whitening causes the separation of fibers off the matrix interface and leads to delamination. Together with this, matrix cracks are formed between the matrix layers as the specimens progressed and the first leakage occurs. As the internal pressure keeps increasing, the leakage turns into an oil jet and ultimate failure occurs when the tubes fail catastrophically [11].

Hashin damage for the composite including fiber tension (HSNFTCRT), fiber compression (HSNFCCRT), matrix tension (HSNMTCRT), and matrix compression ((HSNMCCRT) was obtained. The aim of using this criterion is to identify the status of the stress components at which failure happens. Failure of the composite pipe is usually initiated by matrix cracking. The failure propagates with additional matrix tensile failure (HSNMTCRT) as a result of increasing the cycles, causing a drop in the internal pressure reading which stops the loading processes. In all the cases, matrix tensile failure happened early, before other failure modes, and then the composite pipe is out-of-service, and the modeling was stopped. Other preparties were recorded when the predicted failure reached the value of one as shown in Table 7. It is worth remarking that the HSNMTCRT distribution gives the matrix crack location and DAMAGEMT gives the shape and size of the damage.

**Figure 7:**
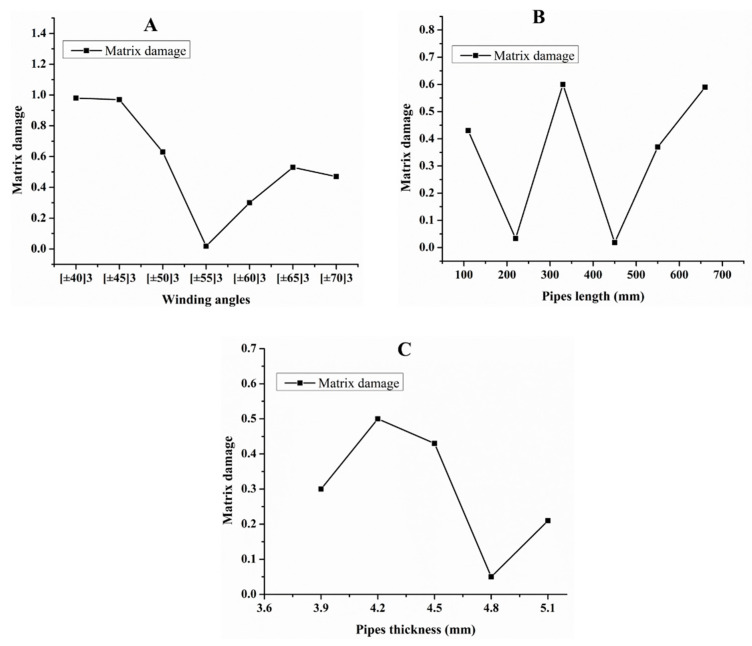
Matrix tensile damage (DAMGEMT) of GFRP composite pipes with varied winding angles (**A**), thicknesses (**B**), and lengths (**C**).

From Table 7, the highest damage size occurred compared to other structures in [±40]_3_ and [±45]_3_, due to fiber overlaps which increased the distance between the fibers and created gaps in the pipe. The maximum pressure that the pipe could handle was 17 for [±40]_3_ and 27 bars for [±45]_3_. After that pressure, matrix cracking was located on the outer surface of the pipe, as seen in Table 7. The [±55]_3_ stacking sequence showed the lowest damage compared to other winding angles. Damage can happen when any of the failure indices reach one. The failure will be due to matrix cracking and then the pipe will lose its function due to leakage. In Table 7, damage in all the cases happened and is shown in the DAMGEMT photos. It is known that for the existence of matrix tension failure predicted by Hashin damage, the value must reach one to prove that the failure happened; otherwise, if the value is bigger than one, it means the failure continues to occur in the structure until the final cracking. Thus, in our case, it was observed that all the values reached one, which means failure happened at a different location in the pipe structure based on pipe specifications, as seen in the matrix tensile damage (DAMGEMT) photos in Table 7 and Figure 7.

## 6. Conclusions

Based on the simulation of the GFRP composite pipe model with different winding angles, pipe lengths, and pipe thicknesses, all the models were subjected to hydrostatic internal pressure, and a failure analysis of the pipe was carried out according to the Hashin for composite failure theory. The effect of winding angles, the length-to-diameter (L/D) and diameter-to-thickness (D/T) ratio, and the failure mechanisms of the composite pipe have been discussed and compared to other structures. The results were the following:

The highest pressure capacity of the composite pipe was shown in [±55]_3_ and this was in agreement with experimental results. In addition, pressure capacity showed an increase with pipe thickness from 3.78 to 5.1 mm.

When the pipe length to diameter (L/D) ratio increases, pressure capacity will decrease.

The effects of the decreased diameter-to-thickness (D/T) ratio led to a decreasing pressure capacity. Other properties did not show a significant issue as the thickness variation was not large.

Matrix tensile failure was the main concern for pipe failure detection, and then the pipe would lose its function due to leakage and structural damage in a specified location.

The average total deformation for the 18 models 0.37 mm for all cases was.

As a recommendation for further studies on the behavior of composite pipes subjected to internal pressure, the following considerations should be given more concern:

The long-term durability of composite pipes under various severe environmental conditions remains unclear. More research should be conducted to investigate the degradation mechanism of composite pipes.

To study their buckling and crushing behavior, more studies on simulated environments should be conducted to fill the knowledge gap on the long-term performance of composite pipes on the seabed and under deep soil when exposed to external hydrostatic pressure.

The degradation of interfacial bonding at the micro-scale in composite pipes urgently needs to be investigated using various methods, because it is a crucial factor influencing the safety and service life of the composite pipes’ structures.

## Figures and Tables

**Table 1:** Comparison of basic physical and mechanical properties between FRP materials and steel [23].

Material Type	Density (g/cm^3^)	Longitudinal Coefficient of Linear Expansion (10–6/°C)	Tensile Strength (MPa)	Young’s Modulus (GPa)	Ultimate Elongation %
GFRP	1.25–2.10	6.0–10.0	483–1600	35–51	1.2–3.1
CFRP	1.50–1.60	−9.0–0.0	600–3690	120–580	0.5–1.7
AFRP	1.25–1.40	−6.0–2.0	1720–2540	41–125	1.9–4.4
* BFRP	1.90–2.10	9.0–12.0	600–1500	50–65	1.2–2.6
Steel	7.85	11.7	483–690	200	6.0–12.0

* BFRP Basalt-fiber-reinforced polymer.

**Table 2:** Composite pipes subjected to internal and external hydrostatic with various parameters and properties.

Composite Pipe	Type of Hydrostatic Pressure	Stacking Sequences	Pressure Capacity (bar)	Failure Modes	Ref.
GRE pipe	Exp. Internal	[±55]_3_	56	Initial leakage and matrix cracking	[11]
GRE pipe	Exp. Internal	[±54_2_/90_8_]	150	Matrix cracking, fiber breakage,	[50]
GRE pipe CF/epoxy (Carbon fiber/epoxy)	Exp. Internal Exp. Internal	[±55/±55] [±50/±50]	80 62	Delamination, fiber, and matrix crack	[51]
FFRE (flax fabric-reinforced epoxy)	Exp. Internal	[0/90]_3_ [0/90]_4_	58 75	Fiber breakage, leakage, and a burst of pipe structures	[52]
Hybrid pipes Carbon/Glass/Glass (CGG)	Exp. Internal	[±55]_3_	32	Matrix cracking, radial cracks, delamination, splitting, and leakage	[53]
Steel and carbon fiber pipe	EXP. and FE. Internal	[0/90]_4_	152	Bulging and rupture	[54]
CF/epoxy	EXP. and FE. Internal	[±75/0] and [±15/0]	63	Fiber and matrix crack	[55]

**Table 3:** The ply properties of GFRP composite being used [11,56].

Property	Value	Property	Value
Longitudinal Young’s moduli, *E*_11_ (MPa)	66,400	Longitudinal tensile strength, *X_t_* (MPa)	700
Transverse Young’s moduli, *E*_22_ (MPa)	12,500	Longitudinal compressive strength, *X_c_* (MPa)	79
Out-of-plane Young’s moduli, *E*_33_ (MPa)	12,500	Transverse tensile strength, *Y_t_* (MPa)	79
Poisson’s ratio, *ν*_12_	0.31	Transverse compressive strength, *Y_c_* (MPa)	65
Poisson’s ratio, *ν*_13_	0.38	Longitudinal shear strength, *S*_12_ (MPa)	50
Poisson’s ratio, *ν*_23_	0.38	Transverse shear strength, *S*_23_ (MPa)	50
Shear modulus, *G*_12_ (MPa)	4520	Density, ρ (kg/m^3^)	1724
Shear modulus, *G*_13_ (MPa)	4550	Thickness, h (mm)	0.63
Shear modulus, *G*_23_ (GPa)	4550	Fiber volume fraction (%)	49.8

**Table 4:** Stacking sequences of GFRP composite pipes being used.

No	Stacking Sequences of Composite Pipe	Winding Angle Degree	Number of Layers
1	[±40]_3_ = [+40, −40, +40, −40, +40, −40]	40	6
2	[±45]_3_= [+45, −45, +45, −45, +45, −45]	45	6
3	[±50]_3_= [+50, −50, +50, −50, +50, −50]	50	6
4	[±55]_3_= [+55, −55, +55, −55, +55, −55]	55	6
5	[±60]_3_= [+60, −60, +60, −60, +60, −60]	60	6
6	[±65]_3_= [+65, −65, +65, −65, +65, −65]	65	6
7	[±70]_3_= [+70, −70, +70, −70, +70, −70]	70	6

**Table 5:** GFRP pipe and simulation input/output parameters.

Specimen Type	Wall Thickness mm	Pipe Length	Hoop Stress MPa	Axial Stress MPa	Hoop to Axial Ratio	Internal Pressure MPa	Pressure Capacity (Bar)	Number of Elements	Study Effect
[±40]_3_	3.78	450	30.2	14.9	2:1	10	17	4408	Effect of winding angles (θ)
[±45]_3_	3.78	450	58.6	28.5	2:1	10	26		
[±50]_3_	3.78	450	90.3	46.2	1.96:1	10	38.2		
[±55]_3_	3.78	450	164.6	82.7	2:1	10	57.2		
[±60]_3_	3.78	450	154.5	77.5	2:1	10	49		
[±65]_3_	3.78	450	144.4	71.9	2:1	10	44.2		
[±70]_3_	3.78	450	132.7	66.6	2:1	10	41.4		
[±55]_3_	3.78	110	200.2	111.6	1.8:1	10	77	1044	Effect of pipe length to diameter (L/D)
		220	190.4	99.3	1.9:1	10	69	2146	
		330	181.5	93.1	1.95:1	10	60.5	3190	
		450	164.6	82.7	2:1	10	57.2	4408	
		550	175.7	92.5	2:1	10	55.6	5336	
		660	148.4	93	2:1	10	55.2	6438	
[±55]_3_	3.9	450	155.2	77.6	2:1	10	59.2	4408	Effect of the pipe diameter to thickness (D/T)
	4.2	450	145.18	72.6	2:1	10	64.7		
	4.5	450	136.38	68.19	2:1	10	71.2		
	4.8	450	128.68	64.34	2:1	10	76.5		
	5.1	450	118.98	59.49	2:1	10	83.1		

**Table 6:** Composite pipe specifications and simulation results of various stress.

Specimen Type	Wall Thickness mm	Pipe Length	S. Mises Stress MPa	Longitudinal Stress (*S*_11_) MPa	Transverse Stress (*S*_22_) MPa	Study Effect
[±40]_3_	3.78	450	32.2	35.4	11.2	Effect of winding angles
[±45]_3_	3.78	450	58.3	62.9	13.3	
[±50]_3_	3.78	450	99.5	107.5	23.8	
[±55]_3_	3.78	450	183.7	196.7	45	
[±60]_3_	3.78	450	150.3	160.2	43.5	
[±65]_3_	3.78	450	167.9	181.5	40.1	
[±70]_3_	3.78	450	136.1	143.6	48.5	
[±55]_3_	3.78	110	281.5	300	44.4	Effect of pipe length to diameter (L/D)
		220	204.4	220.4	47.5	
		330	205.3	220.4	47.8	
		450	183.7	196.7	45	
		550	182.4	197.2	44	
		660	177.2	191.7	43	
[±55]_3_	3.9	450	189.2	204.3	45.2	Effect of diameter to thickness (D/T)
	4.2	450	193.7	209	46	
	4.5	450	197.2	212.7	46.8	
	4.8	450	192.5	207.7	45.7	
	5.1	450	192.6	207.9	45.7	

**Table 7:** Composite pipe matrix cracking and total deformation.

Specimen Type	Wall Thickness mm	Pipe Length	Matrix Tension Failure (HSNMTCRT) and DAMAGEMT	Deformation (mm)
[±40]_3_	3.78	450	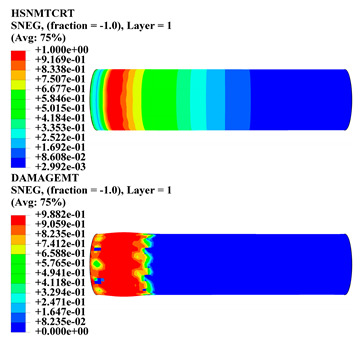	0.38
[±45]_3_	3.78	450	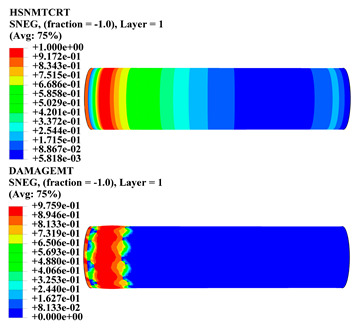	0.38
[±50]_3_	3.78	450	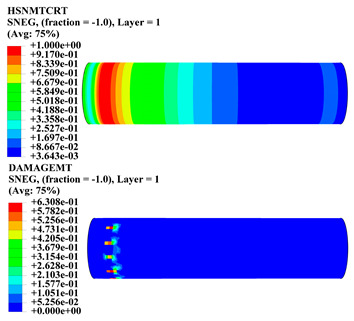	0.37
[±55]_3_	3.78	450	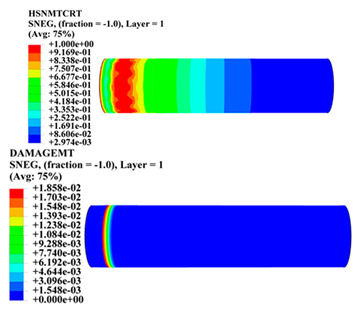	0.39
[±60]_3_	3.78	450	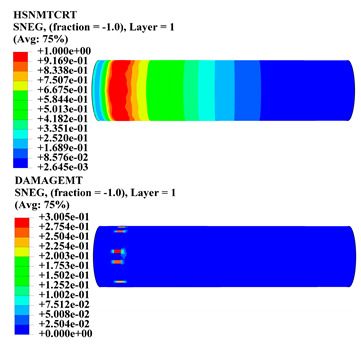	0.39
[±65]_3_	3.78	450	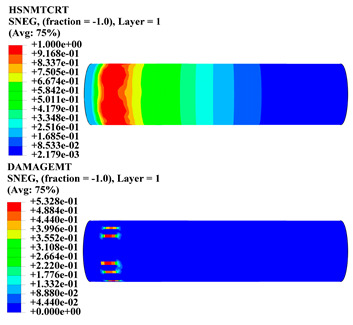	0.42
[±70]_3_	3.78	450	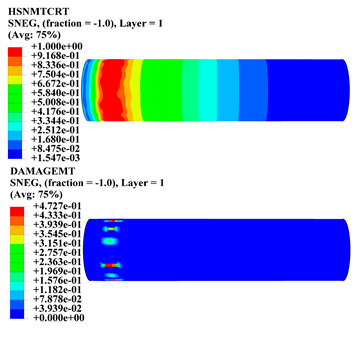	0.40
[±55]_3_	3.78	110	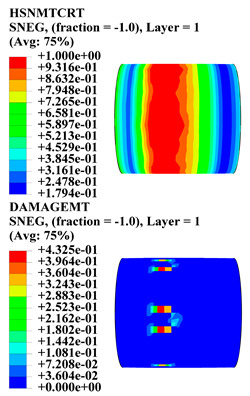	0.39
		220	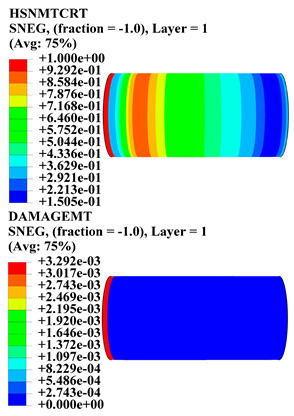	0.31
		330	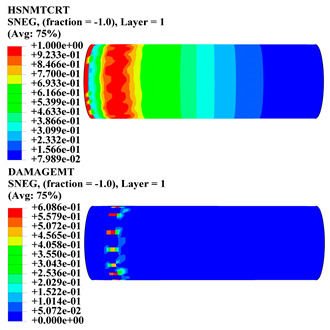	0.37
		450	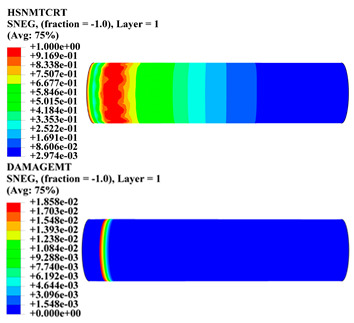	0.39
		550	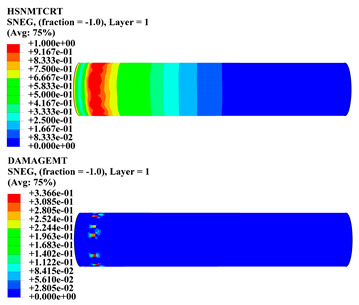	0.37
		660	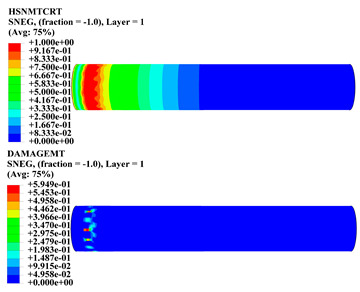	0.38
[±55]_3_	3.9	450	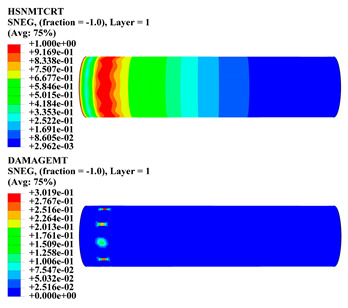	0.35
	4.2	450	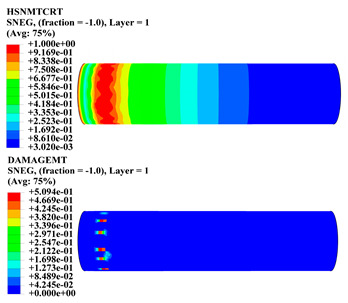	0.36
	4.5	450	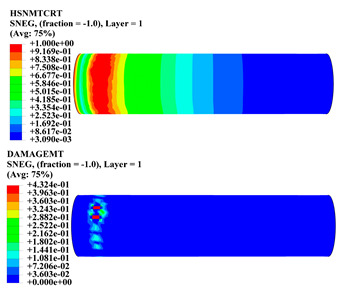	0.38
	4.8	450	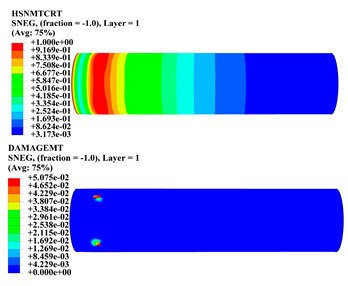	0.35
	5.1	450	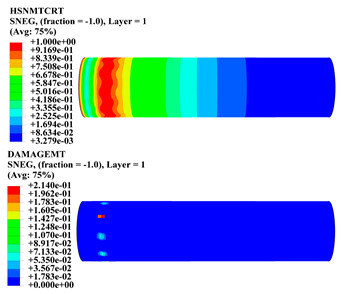	0.35

## Data Availability

Data can be available upon request.

## References

[1] Bakaiyan H., Hosseini H., Ameri E. (2009). Analysis of multi-layered filament-wound composite pipes under combined internal pressure and thermomechanical loading with thermal variations. Compos. Struct..

[2] Xia M., Takayanagi H., Kemmochi K. (2001). Analysis of multi-layered filament-wound composite pipes under internal pressure. Compos. Struct..

[3] Thirumavalavan K., Sarukasan D. (2022). Experimental investigation on multi-layered filament wound basalt/E-glass hybrid fiber composite tubes. Mater. Res. Express.

[4] Prabhakar M.M., Rajini N., Ayrilmis N., Mayandi K., Siengchin S., Senthilkumar K., Karthikeyan S., Ismail S.O. (2019). An overview of burst, buckling, durability and corrosion analysis of lightweight FRP composite pipes and their applicability. Compos. Struct..

[5] Majid M.S.A., Afendi M., Daud R., Amin N., Mohamad A., Cheng E., Gibson A., Hekman M. (2014). General lifetime damage model for glass fibre reinforced epoxy (GRE) composite pipes under multiaxial loading. Key Eng. Mater..

[6] Abdellah M., Alfattani R., Alnaser I., Abdel-Jaber G. (2021). Stress Distribution and Fracture Toughness of Underground Reinforced Plastic Pipe Composite. Polymers.

[7] Gao D., Yang H., Yu W., Wu X., Wu A., Lu G., Zheng Q. (2022). Research on the Mechanical Behavior of Buried Double-Wall Corrugated Pipes. Polymers.

[8] Fekete J.R., Sowards J.W., Amaro R.L. (2015). Amaro, Economic impact of applying high strength steels in hydrogen gas pipelines. Int. J. Hydrog. Energy.

[9] Alamri A.H. (2020). Localized corrosion and mitigation approach of steel materials used in oil and gas pipelines–An overview. Eng. Fail. Anal..

[10] Taheri F. (2013). Advanced fiber-reinforced polymer (FRP) composites for the manufacture and rehabilitation of pipes and tanks in the oil and gas industry. Advanced Fibre-Reinforced Polymer (FRP) Composites for Structural Applications.

[11] Sebaey T.A. (2019). Design of oil and gas composite pipes for energy production. Energy Procedia.

[12] Alabtah F.G., Mahdi E., Eliyan F.F. (2021). The use of fiber reinforced polymeric composites in pipelines: A review. Compos. Struct..

[13] Aljuboury M., Rizvi J., Grove S., Cullen R. (2021). Manufacturing and development of a bolted GFRP flange joint for oil and gas applications. Proc. Inst. Mech. Eng. Part B J. Eng. Manuf..

[14] Kim Y.J. (2019). State of the practice of FRP composites in highway bridges. Eng. Struct..

[15] Siwowski T., Rajchel M., Kaleta D., Własak L. (2017). The first Polish road bridge made of FRP composites. Struct. Eng. Int..

[16] Motlagh B., Gholipour Y., Ebrahimi G. (2012). Experimental investigation on mechanical properties of old wood members reinforced with frp composite. Wood Res..

[17] Selvaraju S., Ilaiyavel S. (2011). Applications of composites in marine industry. J. Eng. Res. Stud..

[18] Vizentin G., Glujić D., Špada V. (2021). Effect of Time-Real Marine Environment Exposure on the Mechanical Behavior of FRP Composites. Sustainability.

[19] Bergan P.G., Buene L., Echtermeyer A.T., Hayman B. (1994). Assessment of FRP sandwich structures for marine applications. Mar. Struct..

[20] Garcia-Espinel J., Castro-Fresno D., Gayo P.P., Ballester-Muñoz F. (2015). Effects of sea water environment on glass fiber reinforced plastic materials used for marine civil engineering constructions. Mater. Des..

[21] Velázquez J.C., Hernández-Sánchez E., Terán G., Capula-Colindres S., Diaz-Cruz M., Cervantes-Tobón A. (2022). Probabilistic and Statistical Techniques to Study the Impact of Localized Corrosion Defects in Oil and Gas Pipelines: A Review. Metals.

[22] Mokhtari M., Melchers R.E. (2018). A new approach to assess the remaining strength of corroded steel pipes. Eng. Fail. Anal..

[23] Dong Z., Wu G. (2019). Research progress on durability of FRP bars reinforced concrete structures. Tumu Gongcheng Xuebao/China Civ. Eng. J..

[24] Bhatt A.T., Gohil P.P., Chaudhary V. (2018). Primary manufacturing processes for fiber reinforced composites: History, development & future research trends. IOP Conf. Ser. Mater. Sci. Eng..

[25] Fred Nilson. What is composite pipe and how is it made?. https://www.linkedin.com/pulse/what-composite-pipe-how-made-fred-nilson/.

[26] Wang S., Wang W., Huang Y., Li S. (2017). The glass coated composite pipe applied in nuclear power plants. E-J. Adv. Maint..

[27] Zubail A., Traidia A., Masulli M., Vatopoulos K., Villette T., Taie I. (2021). Carbon and energy footprint of nonmetallic composite pipes in onshore oil and gas flowlines. J. Clean. Prod..

[28] Krishnan P., Majid M.S.A., Afendi M., Gibson A., Marzuki H. (2015). Effects of winding angle on the behaviour of glass/epoxy pipes under multiaxial cyclic loading. Mater. Des..

[29] Pavlopoulou S., Roy S.S., Gautam M., Bradshaw L., Potluri P. (2017). Numerical and experimental investigation of the hydrostatic performance of fibre reinforced tubes. Appl. Compos. Mater..

[30] Rafiee R., Amini A. (2015). Modeling and experimental evaluation of functional failure pressures in glass fiber reinforced polyester pipes. Comput. Mater. Sci..

[31] Pranesh K., Majid M.A., Afendi M., Marzuki H., Kazim M.N.F.M. (2014). Short-Term Test of±55° Filament Wound GRE Composite Pipes under Multiaxial Stress Ratios. Appl. Mech. Mater..

[32] Fyrileiv O., Venås A. Finite element simulation of pipe-in-pipe systems installed on an uneven seabed. Proceedings of the 34thInternational Conference on Ocean, Offshore and Arctic Engineering.

[33] Rafiee R., Reshadi F. (2014). Simulation of functional failure in GRP mortar pipes. Compos. Struct..

[34] Liu R., Xiong H., Wu X., Yan S. (2014). Numerical studies on global buckling of subsea pipelines. Ocean. Eng..

[35] Assaleh T.A., Almagguz L.A. (2014). Ultimate Elastic Wall Stress (UEWS) Test under Biaxial Loading for Glass-Fibre Reinforced Epoxy (GRE) Pipes. Adv. Mater. Res..

[36] Mattos H.D.C., Reis J., Amorim F., Brandao J., Lana L., Perrut V. (2021). Long-term field performance of a composite pipe repair under constant hydrostatic pressure. Eng. Fail. Anal..

[37] Perillo G., Vedivik N., Echtermeyer A. (2015). Numerical and experimental investigation of impact on filament wound glass reinforced epoxy pipe. J. Compos. Mater..

[38] Mistry J. (1992). Theoretical investigation into the effect of the winding angle of the fibres on the strength of filament wound GRP pipes subjected to combined external pressure and axial compression. Compos. Struct..

[39] Azeem M., Ya H.H., Alam M.A., Kumar M., Stabla P., Smolnicki M., Gemi L., Khan R., Ahmed T., Ma Q. (2022). Application of filament winding technology in composite pressure vessels and challenges: A review. J. Energy Storage.

[40] Guz I.A., Menshykova M., Paik J. (2017). Thick-walled composite tubes for offshore applications: An example of stress and failure analysis for filament-wound multi-layered pipes. Ships Offshore Struct..

[41] Huang Z., Qian X., Su Z., Pham D.C., Sridhar N. (2020). Experimental investigation and damage simulation of large-scaled filament wound composite pipes. Compos. Part B Eng..

[42] Perillo G., Vacher R., Grytten F., Sørbø S., Delhaye V. (2014). Material characterisation and failure envelope evaluation of filament wound GFRP and CFRP composite tubes. Polym. Test..

[43] Rafiee R. (2016). On the mechanical performance of glass-fibre-reinforced thermosetting-resin pipes: A review. Compos. Struct..

[44] GÜNÖZ A., Kepir Y., Memduh K. (2022). The investigation of hardness and density properties of GFRP composite pipes under seawater conditions. Turk. J. Eng..

[45] Rafiee R., Habibagahi M. (2018). Evaluating mechanical performance of GFRP pipes subjected to transverse loading. Thin-Walled Struct..

[46] Gunoz A., Kepir Y., Kara M. (2020). Tensile strength alteration of GFRP composite pipes under seawater-dominated conditions. J. Fail. Anal. Prev..

[47] Saghir F., Gohari S., Mozafari F., Moslemi N., Burvill C., Smith A., Lucas S. (2021). Mechanical characterization of particulated FRP composite pipes: A comprehensive experimental study. Polym. Test..

[48] Hawa A., Majid M.A., Afendi M., Haslan M., Pranesh K., Amin N. (2015). Burst strength of glass fibre/epoxy composite pipes subjected to impact loading. Appl. Mech. Mater..

[49] Krishnan P., Majid M.A., Afendi M., Yaacob S., Gibson A. (2016). Effects of hydrothermal ageing on the behaviour of composite tubes under multiaxial stress ratios. Compos. Struct..

[50] Rafiee R., Torabi M., Maleki S. (2018). Investigating structural failure of a filament-wound composite tube subjected to internal pressure: Experimental and theoretical evaluation. Polym. Test..

[51] Maziz A., Rechak S., Tarfaoui M. (2021). Comparative study of tubular composite structure subjected to internal pressure loading: Analytical and numerical investigation. J. Compos. Mater..

[52] Firouzsalari S.E., Dizhur D., Jayaraman K., Ingham J. (2021). Experimental study of flax fabric-reinforced epoxy pipes subjected to internal pressure. Compos. Part A Appl. Sci. Manuf..

[53] Gemi L. (2018). Investigation of the effect of stacking sequence on low velocity impact response and damage formation in hybrid composite pipes under internal pressure. A comparative study. Compos. Part B Eng..

[54] Zeng W., Zhao H., Wang Y., Li Y., Huo S. (2020). Hydrostatic Test and Simulation Verification of Pipeline with Carbon Fiber Repair. IOP Conf. Series Earth Environ. Sci..

[55] Sohn J.M., Hirdaris S., Romanoff J., Kim S.J. (2022). Development of Numerical Modelling Techniques for Composite Cylindrical Structures under External Pressure. J. Mar. Sci. Eng..

[56] Khashaba U., Sebaey T., Mahmoud F., Selmy A., Hamouda R., Sebaey T. (2013). Experimental and numerical analysis of pinned-joints composite laminates: Effects of stacking sequences. J. Compos. Mater..

[57] Pavlou D.G. (2013). Composite Materials in Piping Applications: Design, Analysis and Optimization of Subsea and Onshore Pipelines from FRP Materials.

[58] Grove S. (1999). Optimum fiber orientation in filament wound structures. J. Mater. Sci. Lett..

[59] Sülü İ.Y. (2016). Stress analysis of multi-layered hybrid composite pipes subjected to internal pressure. Int. J. Eng. Appl. Sci..

[60] Velázquez J.C., González-Arévalo N.E., Díaz-Cruz M., Cervantes-Tobón A., Herrera-Hernández H., Hernández-Sánchez E. (2022). Failure pressure estimation for an aged and corroded oil and gas pipeline: A finite element study. J. Nat. Gas Sci. Eng..

